# 2022 ACVIM Forum Research Report Program

**DOI:** 10.1111/jvim.16538

**Published:** 2022-10-12


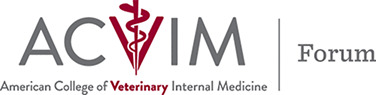
The American College of Veterinary Internal Medicine (ACVIM) Forum and the Journal of Veterinary Internal Medicine (JVIM) are not responsible for the content or dosage recommendations in the abstracts. The abstracts are not peer reviewed before publication. The opinions expressed in the abstracts are those of the author(s) and may not represent the views or position of the ACVIM. The authors are solely responsible for the content of the abstracts.

**2022 ACVIM Forum**

**June 22 – October 31, 2022**

**Research Report Program**

**Index of Abstracts**

**Table:** 

**THURSDAY, JUNE 23**		
Time	Presenting Author	Abstract Title
**CARDIOLOGY**		
4:30 pm	Clarke Atkins	Mitral Regurgitation Severity Index: A Simple Calculation for Classification and Prognostication?
5:00 pm	Lauren Markovic	Utility of 3‐Dimensional Models in Canine Congenital Heart Disease
5:30 PM	Min Su Kim	Clinical Application of Novel Heartworm Removal Forceps
**NEUROLOGY**		
10:00 am	Vishal Murthy	Long‐term Outcomes of Canine Calvarial Multilobular Tumor of Bone Treated by Craniectomy
10:30 am	Lynette Cole	Normative Brainstem Auditory Evoked Responses in Cavalier King Charles Spaniels with Chiari‐like Malformation
11:15 am	Tom Jukier	Single Dose Pharmacokinetics of an FDA Approved Cannabidiol Medication in Healthy Cats
**FOOD ANIMAL INTERNAL MEDICINE**		
1:45 pm	Chelsea Holschbach	Prognostic Indicators for Survival of Downer Cows Treated with Use of a Flotation Tank
2:15 pm	Maria Puerto‐Parada	Effects of Blood Contamination on Total Nucleated Cell Counts and Protein Concentrations in Bovine CSF
**FRIDAY, JUNE 24**		
Time	Presenting Author	Abstract Title
**ONCOLOGY**		
8:00 AM	Audrey Ruple	Lifetime Prevalence of Tumors in Companion Dogs Included in the Dog Aging Project Baseline Data
8:30 AM	Esther Chon	Utility of Tumor Genomic Analysis for Diagnostically Challenging Cancer Cases
9:15 AM	Noe Reyes	Cytokine Expression of Activated T cells in Dogs Undergoing Adoptive T cell therapy for Osteosarcoma
9:45 am	Heather Wilson‐Robles	Evaluation of Plasma Nucleosome Concentrations in Healthy Dogs and Dogs with Various Common Cancers
**SMALL ANIMAL INTERNAL MEDICINE**		
8:00 AM	Gerald Beddies	Evaluation of Pharmacodynamic Effects of a Hypoxia‐Inducible‐Factor Prolyl Hydroxylase Inhibitor on Erythropoiesis of Healthy Cats
8:30 AM	Emily Gould	Esomeprazole Alters Neoplastic Canine Mast Cell Structure, Viability, and Function
9:15 AM	Karin Allenspach	Decreased Duodenal Enterocyte Height and Width is a Hallmark of Chronic Enteropathy in Dogs
**EQUINE**		
8:00 AM	Kate Hepworth‐Warren	Risk Factors Associated with an Equine Coronavirus Outbreak at a Saddlebred Farm in North Carolina
8:30 AM	Fairfield Bain	Voluntary Surveillance Program for Equine Influenza Virus in the United States from 2008 ‐ 2021
9:15 AM	Sharanne Raidal	Antibiogram Use to Inform Antimicrobial Selection in Equine Practice
9:45 AM	Jenni Bauquier	Pharmacokinetics and Ex‐Vivo Pharmacodynamics of the Novel Anti‐Inflammatory Drug, Doramapimod, in Horses
11:00 AM	Kari Bevevino	Feasibility of a Point of Care Ultrasound Protocol for Cardiorespiratory Evaluation in Horses
11:30 AM	Sharanne Raidal	Effects of Non‐invasive Ventilation on Respiratory Function and Lung Volume in Foals
**ON DEMAND**		
Presenting Author	Abstract Title	
**SMALL ANIMAL INTERNAL MEDICINE**		
Chen Gilor	Once‐daily Insulin Glargine 300 U/ml for the Treatment of Canine Diabetes Mellitus	

## 1 CARDIOLOGY

## 2 MITRAL REGURGITATION SEVERITY INDEX: A SIMPLE CALCULATION FOR CLASSIFICATION AND PROGNOSTICATION?

### 2.1 **Clarke E. Atkins** ^1^; Darcy Adin^2^, DVM, DACVIM (Cardiology); Thomas Blondell^3^, PhD; Emilie Guillot^4^, DVM; Michelle Vereb^5^, DVM; Jessica Ward^6^, DVM, DACVIM (Cardiology)

#### 2.1.1 ^1^North Carolina State University; ^2^Clinical Professor, Large Animal Clinical Sciences, University of Florida; ^3^CEVA Santé Animale; ^4^Global Technical Director, CEVA Santé Animale; ^5^Iowa State University; ^6^Associate Professor, Iowa State University

**background:** Prognosis in myxomatous mitral valve disease (MMVD) is variable. Better methods of predicting outcome would aid in communication with individual pet owners and sub‐division of research cohorts. The mitral regurgitation severity index (MRSI) was originally developed by VETPROOF (Veterinary Enalapril Trial to Prove Reduction in Onset Of Failure) investigators to predict MMVD outcome. While other promising indices are now available, MRSI is appealing in its simplicity. This abstract serves as the seminal report of MRSI's efficacy in prognosticating asymptomatic MMVD.

**hypothesis/objectives:** MRSI will predict time to CHF.

**animals:** 133 dogs with ACVIM Stage B2 MMVD.

**methods:** VETPROOF entry data from dogs randomized to either enalapril or placebo were retrospectively evaluated. Potentially useful indicators of time to onset of heart failure (CHF), including age, heart rate, and echocardiographically‐determined left atrium‐to‐aortic ratio (LA:Ao), were used to establish MRSI, by the following equation:

MRSI = [(Age/10) x (Heart‐Rate/120) x (LA:Ao)] x 100

Kaplan‐Meier survival curves and Cox proportional hazard analyses were used to evaluate the predictive ability of MRSI for time to CHF (significance, *P*<0.05).

**results:** MRSI, stratified at <150, 150–249, and >249 demonstrated significant differences in time to the endpoints, thereby predicting MMVD prognosis (Figure).

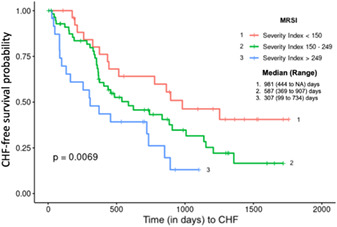

**conclusion:** MRSI is easily obtained and useful in stratification and prognostication of MMVD. Studies are ongoing to test this index more rigorously in a larger cohort of dogs with MMVD, stage B2 or C. The MRSI could be modified by substituting radiographic determination of LA:Ao, making calculation and utilization of MRSI more practitioner‐friendly.

## 3 UTILITY OF 3‐DIMENSIONAL MODELS IN CANINE CONGENITAL HEART DISEASE

### 3.1 **Lauren E. Markovic** ^1^; Amanda Coleman^2^, DVM, DACVIM (Cardiology); Brian Sutherland^3^, DVM, DACVS (SA)

#### 3.1.1 ^1^University of Georgia; ^2^Associate Professor, Small Animal Medicine and Surgery, University of Georgia; ^3^Assistant professor, Small Animal Medicine and Surgery, University of Georgia

**background:** Three‐dimensional (3D) cardiac modeling of congenital heart disease (CHD) has shown promise in human medical applications, particularly for pre‐procedural planning, simulation, and education. Utilization of 3D CHD models in veterinary medicine deserves further investigation.

**hypothesis/objectives:** This study sought to develop a protocol to generate 3D models of naturally occurring canine congenital heart disease from computed tomography scans. We hypothesized that cardiac image‐based engineering software could be used to create canine‐specific 3D models for pre‐procedural planning and education.

**animals:** Five client‐owned dogs diagnosed with CHD considered amenable to transcatheter or surgical intervention.

**methods:** This prospective study utilized digital datasets from five dogs with CHD that underwent computed tomography angiography. Congenital heart diseases included patent ductus arteriosus (1), pulmonary valve stenosis with ventricular septal defect (1), atrial septal defect (1), patent ductus arteriosus with persistent left cranial vena cava (1) and tetralogy of Fallot (1). Segmentation and computer‐aided design of digital datasets was performed with Materialise Mimics 24.0 and Materialise 3‐matic 16.0 (Materialise NV, Leuven, Belgium). A cardiovascular tool, known as CT‐Heart, was used to provide high‐quality segmentation of cardiac chambers and great vessels.

**results:** Three‐dimensional models were created in all dogs, allowing spatial conceptualization of cardiovascular structures. Three of five dogs went on to have transcatheter cardiac intervention (patent ductus arteriosus occlusion) or palliative surgery (modified Blalock‐Taussig shunt). All 3D models were used for either veterinary clinical education or pre‐procedural planning.

**conclusions:** This study demonstrated that canine‐specific 3D models of CHD can be created using cardiovascular modeling software.

## 4 CLINICAL APPLICATION OF NOVEL HEARTWORM REMOVAL FORCEPS

### 4.1 **Min Su Kim** ^1^; Meg Sleeper^2^, VMD, DACVIM (Cardiology); Darcy B. Adin^3^, DVM, DACVIM (Cardiology); Michael Aherne^4^, MVB, GradDipVetStud, MS, MANZCVS (Small Animal Surgery), DACVIM (Cardiology); Amara H. Estrada^5^, DVM, DACVIM (Cardiology); Daesik Kim^6^, DVM, MS; Daeyun Seo^6^, DVM

#### 4.1.1 ^1^College of Veterinary Medicine, Seoul National University; ^2^Clinical Professor, Small Animal Clinical Sciences, College of Veterinary Medicine, University of Florida; ^3^Clinical Professor, Large Animal Clinical Sciences, College of Veterinary Medicine, University of Florida; ^4^Clinical Assistant Professor, Small Animal Clinical Sciences, College of Veterinary Medicine, University of Florida; ^5^Professor, Small Animal Clinical Sciences, College of Veterinary Medicine, University of Florida; ^6^Clinical Veterinarian, Department of Clinical Science, College of Veterinary Medicine, Seoul National University

**background:** Mechanical removal of heartworms from the cardiac chambers and main pulmonary arteries benefits dogs with severe heartworm infection by rapidly reducing worm burden, but there is a need for additional device options to accomplish this.

**hypothesis/objectives:** Our novel heartworm forceps facilitate safe and effective worm removal from dogs.

**animals:** Ten dogs with heartworm infection (Class 3 and 4).

**methods:** We retrospectively evaluated the use of our novel heartworm removal forceps in 10 dogs. The rotatable basket‐shaped forceps have a 45‐degree angle at the tip for easy access to the intracardiac and pulmonary arteries and the removal of many worms at a time. The number of worms pulled and short‐term follow‐up were reported for these 10 dogs.

**Figure:**
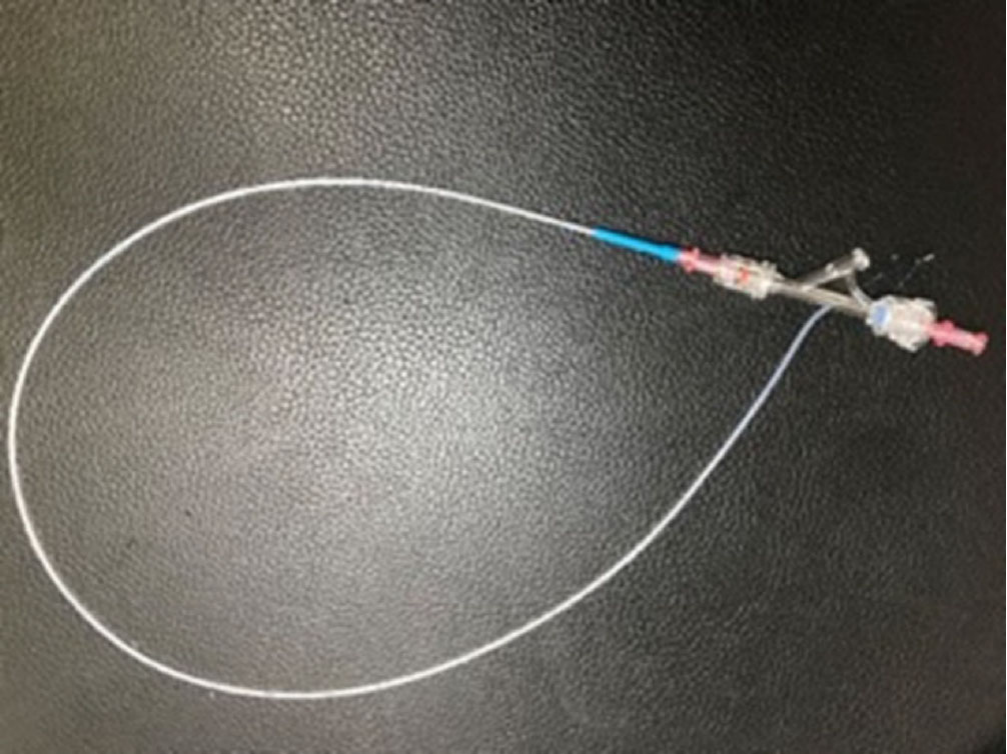
**Figure 1.** Novel heartworm removal forceps

**results:** The procedure was performed under general anesthesia via right jugular venotomy with fluoroscopic guidance. Heartworms were found in the right atrium and right ventricle of all patients, including 1 patient found in the pulmonary artery. All patients had no complications during the removal. The mean (SD) number of removed heartworms was 21±14. No heartworms were detected by echocardiography in the heart and pulmonary arteries immediately and 10 days post‐procedure in 7 dogs. In 3 dogs, 1 or 2 worms were visualized in the pulmonary arteries after the removal.

**Figure:**
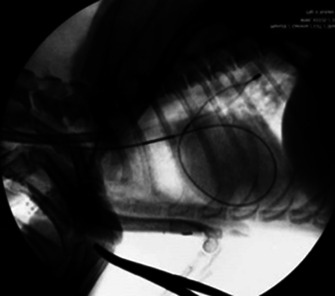
**Figure 2.** Pulmonary artery access under fluoroscopy

**conclusions and clinical importance:** This study showed that these novel forceps can safely remove heartworms from intracardiac and pulmonary arterial locations in dogs with heartworm infection.

## 5 NEUROLOGY

## 6 LONG‐TERM OUTCOMES OF CANINE CALVARIAL MULTILOBULAR TUMOR OF BONE TREATED BY CRANIECTOMY

### 6.1 **Vishal D. Murthy** ^1^; John Rossmeisl^2^, DVM, DACVIM (Neurology); Laura White^3^, DVM, DACVP; John Robertson^2^, VMD, DACVP; John Gay^4^, DVM, DACVPM; Patrick Kenny^5^, DVM, DACVIM (Neurology), DECVN; Annie Chen^4^, DVM, DACVIM (Neurology); Chai‐Fei Li^6^, DVM, DACVIM (Neurology); Peter Dickinson^6^, DVM, DACVIM (Neurology); Karen Vernau^6^; Beverly Sturges^6^, DVM, DACVIM (Neurology)

#### 6.1.1 ^1^Washington State University; ^2^Department of Small Animal Clinical Sciences, Virginia‐Maryland Regional College of Veterinary Medicine, Virginia Polytechnic Institute and State University; ^3^Washington Animal Disease Diagnostic Laboratory, College of Veterinary Medicine, Washington State University; ^4^Department of Veterinary Clinical Sciences, College of Veterinary Medicine, Washington State University; ^5^Small Animal Specialist Hospital; ^6^Department of Surgical and Radiological Sciences, School of Veterinary Medicine, University of California‐Davis

**background:** Calvarial multilobular tumor of bone (MLTB) is typically treated with surgical excision and various adjunctive therapies. However, survival and recurrence outcome data are lacking.

**hypothesis/objectives:** To describe the clinical features, imaging characteristics, surgical treatment, and long‐term outcomes of dogs with calvarial MLTB.

**animals:** 53 client‐owned dogs with histologically confirmed MLTB.

**methods:** Retrospective case series of canine MLTB treated with craniectomy. Signalment, examination findings, advanced imaging findings, surgical technique, histological grade, and outcome (survival, recurrence) were recorded.

**results:** Most dogs showed no neurological deficits (66.0%). Tumors commonly involved the frontal (30.4%) and parietal (29.4%) bones. Venous sinuses were frequently involved on imaging (45.1%) and in surgery (44.2%). Intraoperative complications (e.g., hemorrhage, cerebral swelling) occurred in 24.5% of cases. Postoperative complications, occurring in 43.4% of cases, included aspiration pneumonia, seizures, and intracranial hypertension. Tumors were not routinely graded (56.6% ungraded), limiting evaluation. Incomplete surgical margins were identified on histopathology in 69.8% of cases and had a negative effect on time to recurrence (HR 9.6; 95% CI 1.6–89.3; *P*=0.02), as did a history of preoperative biopsy (HR 4.0; 95% CI 1.150 to 15.93; *P*=0.04). The use of adjunctive therapies had a positive effect on time to recurrence (HR 0.21; 95% CI 0.04–0.9; *P*=0.04). Median survival time was 1088 days (range 0–3165 days) and median time to recurrence was 470 days (range 53–916 days).

**conclusions and clinical importance:** Long‐term remission and survival is possible with calvarial MLTB. Histological grading and margin evaluation should be routinely performed.

## 7 NORMATIVE BRAINSTEM AUDITORY EVOKED RESPONSES IN CAVALIER KING CHARLES SPANIELS WITH CHIARI‐LIKE MALFORMATION

### 7.1 **Lynette K. Cole**; Susan Wagner; Sarah Moore; Ronaldo da Costa; Eric Hostnik; Laura Selmic

#### 7.1.1 College of Veterinary Medicine, The Ohio State University

**background:** Hearing in dogs can be evaluated using brainstem auditory evoked response (BAER) testing. Cavalier King Charles spaniels (CKCS) are prone to Chiari‐like malformation (CM).

**hypothesis/objectives:** To evaluate hearing loss in CKCS, BAER parameters are needed in reference to presence or absence of CM. The purpose was to establish normative BAER data. The specific aim was to determine if BAER indices differed based on CM grade. We hypothesized that CM would result in latency differences in CKCS with CM grade 2 (CM2) compared to those with CM0 and CM1.

**animals:** Twenty CKCS without apparent hearing abnormalities assessed by owner.

**methods:** Prospective case series. Under general anesthesia, CKCS underwent CT scan (assess middle ear), BAER testing, MRI (assess presence/grade of CM).

**results:** Nine (45%) CKCS had CM1; 11 (55%) had CM2. All had at least one morphologic abnormality in waveforms. Absolute and interpeak latencies were reported for all CKCS and compared between CM grades. Median threshold for CKCS with CM1 was 39 and CM2 was 46. Absolute latencies for CKCS with CM2 were consistently longer than those for CKCS with CM1 with exception of Wave II, V at 33 dB. Significant differences were found for wave V at 102 dB (p=0.04), wave II at 74 dB (*p*=0.008). Interpeak latency comparisons were inconsistent between CM1 and CM2.

**conclusions and clinical importance:** Normative BAER data for CKCS with CM1 and CM2 were established. Results suggest CM impacts BAER latency results, but influence is not always statistically significant or predictable.

## 8 SINGLE DOSE PHARMACOKINETICS OF AN FDA‐APPROVED CANNABIDIOL MEDICATION IN HEALTHY CATS

### 8.1 **Tom Jukier** ^1^; Crisanta Cruz‐Espindola^1^ , MS; Dawn Boothe^2^, DVM, MS, PhD, DACVIM, DACVCP; Doug Martin^2^, PhD

#### 8.1.1 ^1^Auburn University; ^2^Professor, Anatomy, Physiology, and Pharmacology, Auburn University

**background:** Cannabidiol (CBD) is emerging as a potential therapeutic agent in human and veterinary medicine for a myriad of conditions. The FDA has recently approved a CBD (Epidiolex®) in humans as add‐on therapy for seizures, where oral absorption is increased in fed versus fasted states. Furthermore, recent evidence associated higher CBD levels with improved seizure control.

**hypothesis/objective:** Determination of a dose of Epidiolex® in healthy cats necessary to achieve anticipated antiseizure therapeutic concentrations, and to determine relative oral bioavailability of fasted versus fed animals. We hypothesized that oral bioavailability would be higher in fed cats.

**animals:** 10 healthy cats from a research colony.

**methods:** On study day, each cat received 5 mg/kg of CBD orally. Cats were randomized to be fed (n=5) or not fed (n=5) approximately 30 minutes prior to oral dosing. Blood samples were collected serially once prior to and following administration for 48 hrs. Following a 1‐month washout, the study was repeated by crossing cats to the alternate fed state.

**results:** Mean maximum plasma concentration (*C*_max_, ng/mL), time to maximum concentration (*T*_max_, hours), disappearance half‐life (hours), area under the curve (ng*h/mL) and mean residence time (hours) for the fasted group were 269±333, 2.58±1.6, 6.3±3.6 hours, 921.5±1003, and 12.1±7.5 respectively, while for the fed group 465±220, 4.7±2.1, 6.9±2.9, 2650±1188, 10.2±2.4, respectively. Significant difference was limited to *T*_max._ Relative bioavailability (*F*) after feeding compared to fasting was 2.9.

**conclusions:** Epidiolex® administered orally appears to reach potentially therapeutic concentrations in healthy cats and oral bioavailability increases with feeding.

## 9 ONCOLOGY

## 10 LIFETIME PREVALENCE OF TUMORS IN COMPANION DOGS INCLUDED IN THE DOG AGING PROJECT BASELINE DATA

### 10.1 **Audrey Ruple** ^1^; Stephen Schwartz^2^; Silvan Urfer^3^; Michelle White^4^; Kate Megquier^4^; Sandi Shrager^3,5^

#### 10.1.1 ^1^Virginia Tech; ^2^Fred Hutchinson Cancer Research Center; ^3^University of Washington; ^4^Broad Institute; ^5^The Dog Aging Project Consortium—The Dog Aging Project

**background:** Cancer is a leading cause of morbidity and mortality in dogs in the United States, yet its frequency has not been robustly characterized. Most reports about cancer outcomes in dogs comes from populations seen at tertiary care centers. The Dog Aging Project (DAP), a longitudinal study of companion dogs in the US, provides an opportunity for cancer outcomes in dogs to be described more completely and associations between risk factors and outcomes to be investigated.

**objective:** To estimate the lifetime prevalence of malignant and non‐malignant tumors in companion dogs and investigate associations between dog characteristics and tumor outcomes.

**animals:** 27,541 dogs enrolled in the DAP as of December 31, 2020.

**methods:** Lifetime prevalence was calculated per 1,000 DAP participants. Crude associations were estimated in relation to the dog's current age, breed, and size. Age‐adjusted prevalence ratios (PRs) and corresponding 95% confidence intervals (CI) were estimated using Poisson regression models.

**results:** 1,111 dogs were reported to have a history of malignant (819) or non‐malignant (404) tumors. The lifetime prevalences of malignant and benign tumors were 29.7 and 14.7 per 1000 dogs, respectively. Dogs with a history of tumors (malignant or non‐malignant) were more likely to be older and larger than the DAP population averages. Age‐adjusted lifetime malignancy prevalence ratios increased with increasing dog size.

**conclusions:** We observed the lifetime prevalence of malignant tumors to increase with increasing dog size. Ongoing prospective data collection for DAP will permit more nuanced studies of risk factors for canine tumor incidence.

## 11 UTILITY OF TUMOR GENOMIC ANALYSIS FOR DIAGNOSTICALLY CHALLENGING CANCER CASES

### 11.1 **Esther Chon**; Guannan Wang, PhD; Derick Whitley, DVM, DACVP; Shukmei Wong; Natalie Duran; Jonathan Adkins; Zhanyang Zhu, PhD; Manisha Warrier; Salvatore Facista; David Haworth, DVM, PhD; William Hendricks, PhD

#### 11.1.1 Vidium Animal Health

**background:** Poorly differentiated tumors are clinically challenging since an unclear diagnosis hinders therapeutic planning and prognostication. Therefore, new diagnostic tools are necessary for effective management of these cases. Tumor genomic analysis and mutation interpretation guided by a growing wealth of published data has potential to meet this need by providing clinically actionable information.

**hypothesis/objectives:** Our objective was to determine the diagnostic, prognostic, and therapeutic utility of genomic analysis for cases with ambiguous diagnoses and therefore uncertain treatment plans and unclear prognoses.

**animals:** Thirty‐five clinical oncology cases submitted for tumor genomic analysis with SearchLight DNA™, a genomic tumor profiling panel, were selected for evaluation.

**methods:** Cases with histories or pathology reports containing the words “poorly differentiated”, “anaplastic”, “probable”, “possible”, “suspect”, “suggestive”, “malignant”, “neoplasia/neoplasm” or “round cell tumor” without further description of tumor type, or any non‐specific diagnostic descriptors such as “atypical” were evaluated as a case series. Tumor‐only next‐generation sequencing was performed for all cases to identify mutations with biomarker associations in 120 cancer genes. Mutations with diagnostic, prognostic, or predictive associations were annotated according to our proprietary biomarker database and reported. These genomic reports were reviewed to identify cases in which genomic data provided medically applicable direction.

**results:** In over 80% of these cases, genomic data provided any combination of diagnostic clarity, prognostic guidance, and/or treatment options.

**conclusions and clinical importance:** Genomic analysis is a useful tool for the clinical management of diagnostically challenging cancers that would otherwise be difficult to treat or prognosticate.

## 12 CYTOKINE EXPRESSION OF ACTIVATED T CELLS IN DOGS UNDERGOING ADOPTIVE T CELL THERAPY FOR OSTEOSARCOMA

### 12.1 **Noe Reyes** ^1^; Evan Courtemanche^2^; Jody Ehrhardt^3^, CCRC; Tammie Wahaus^4^

#### 12.1.1 ^1^ELIAS Animal Health; ^2^Vice President of Manufacturing, Manufacturing, ELIAS Animal Health, LLC; ^3^Director of Clinical Trials, ELIAS Animal Health, LLC; ^4^CEO, Executive, ELIAS Animal Health

**background:** Immunotherapeutics have revolutionized how cancer may be treated by conditioning the host immune system to generate an anti‐cancer response. These therapies are becoming more available to veterinary practitioners. Here we examine blood cytokine expression sampled from dogs undergoing vaccine‐enhanced adoptive T cell therapy (VACT).

**hypothesis/objectives:** T cells conditioned in vivo to cancer antigens will, after ex vivo activation and expansion, release large amounts of proinflammatory cytokines. T cells conditioned to cancer antigens, but not activated, will have lesser cytokine expression in the presence of cancer cells.

**animals:** 4 privately owned dogs undergoing VACT for appendicular osteosarcoma.

**methods:** Dogs underwent VACT consisting of 3 weekly autologous cancer cell vaccinations, T cell harvest via apheresis, ex vivo T cell activation and reinfusion. Samples were collected at three timepoints: prior to vaccination, two weeks post‐vaccination, and post‐T cell activation.

**results:** High levels of proinflammatory cytokines (e.g., IFN‐gamma, IL‐6, TNF‐alpha) were expressed by post‐activation T cells in the presence of host tumor cells, whereas naïve T cells and post‐vaccination T cells did not express high levels of these cytokines in the presence of host tumor cells.

**conclusions and clinical importance:** Host T cells conditioned to target cancer antigens via autologous cancer cell vaccination and then ex vivo activated and expanded can actively mount an anti‐cancer immune response. In the presence of the target cancer cells, these activated T cells will initiate an immune response via upregulation of cytokine expression.

## 13 EVALUATION OF PLASMA NUCLEOSOME CONCENTRATIONS IN HEALTHY DOGS AND DOGS WITH VARIOUS COMMON CANCERS

### 13.1 **Heather M.** **Wilson‐Robles** ^1^ ; Thomas Butera^2^, DVM; Thomas Bygott^3^; Jill Jarvis^4^; Theresa Kelly^5^, PhD; Pamela Miller^6^; Tasha Miller^7^, BS; Jason Terrell^8^, MD

#### 13.1.1 ^1^Texas A&M University; ^2^Chief Executive Officer, Volition Veterinary Diagnostic Development, Volition America Inc.; ^3^Bioinformatics Director, VolitionRx; ^4^Medical Oncology Research Technician, VSCS, Texas A&M University; ^5^Chief Science Officer, Volition America Inc.; ^6^Laboratory Technician, VSCS, Texas A&M University; ^7^Laboratory Manager, VSCS, Texas A&M University; ^8^Chief Medical Officer, Volition America Inc.

**background:** Nucleosomes are released into circulation during apoptosis or necrosis in a variety of diseases. Changes in plasma nucleosome concentrations have been reported to be useful epigenetic biomarkers for monitoring treatment response of a variety of cancers in humans. In dogs, elevated plasma nucleosome concentrations have been documented in lymphoma and hemangiosarcoma.

**hypothesis/objectives:** The objective of this study was to quantify and better characterize plasma nucleosomes in dogs with various common malignancies.

**animals:** A total of 662 cancer patients and healthy dogs were included in this study (528 dogs with cancer and 134 healthy dogs).

**methods:** Canine plasma samples from the NCI‐DCTD biorepository and from active patients or healthy volunteers at a Small Animal Teaching Hospital were purchased or collected with owner consent and IACUC approval, respectively, for this study. Samples were processed immediately and then stored at ‐80°C until they could be run in batches. The Nu.Q® H3.1 total nucleosome ELISA assay was performed according to the manufacturer's protocol.

**results:** The most common cancers evaluated included lymphoma (LSA; n=126), hemangiosarcoma (HSA; n=77), osteosarcoma (OSA; n=49), soft tissue sarcoma (STS; 51), malignant melanoma (n=49), mast cell tumors (MCT; n=126) and histiocytic sarcoma (n=26). A receiver operator characteristic curve AUC was 68.7% for all cancers. Cancers with the highest AUCs included hemangiosarcoma (91.74%), lymphoma (87.83%), histiocytic sarcoma (83.01%) and malignant melanoma (75.05%).

**conclusion and clinical importance:** This assay is useful for detecting common cancers that are more systemic or those that have a high cellular turnover rate.

## 14 SMALL ANIMAL INTERNAL MEDICINE

## 15 EVALUATION OF PHARMACODYNAMIC EFFECTS OF A HYPOXIA‐INDUCIBLE FACTOR PROLYL HYDROXYLASE INHIBITOR ON ERYTHROPOIESIS OF HEALTHY CATS

### 15.1 **Gerald Beddies** ^1^; Annette Boegel^1^; Ingo Flamme^2^; Ralph Krebber^2^; Terry Settje^3^; Franziska Schmidt^1^; Eva Kruedewagen^1^; Sandra Mangold‐Gehring^1^ ; Chantal Lainesse^4^, DVM, PhD, DACVCP

#### 15.1.1 ^1^Elanco Animal Health; ^2^Bayer AG; ^3^Olathe, Kansas; ^4^Integral Consulting Strategies, Inc.

**background:** Inhibition of hypoxia‐inducible factor prolyl hydroxylase (HIF‐PH) stimulates erythropoiesis in rats, dogs, monkeys, and humans.

**objective:** Investigation whether molidustat, a novel HIF‐PH inhibitor, stimulates the production of erythrocytes in healthy cats.

**animals:** Seventeen healthy adult research colony cats (1–2 years old).

**methods:** In a randomized, placebo‐controlled pilot laboratory study, cats were treated once daily with oral suspensions of 0 (Group 1; n=6), 5 (Group 2; n=6) or 10 (Group 3; n=5) mg/kg of molidustat. Effects on hematocrit (HCT) and erythropoietin (EPO) concentrations were evaluated weekly. Molidustat treatment was ceased when HCT exceeded 60% at two consecutive measurements.

**results:** Compared to placebo, a statistically significant increase in mean HCT was evident starting on Day 14 (Group 2: 54.4% versus 40.3%, *p*<0.001, 95% CI [8.95–19.28]; Group 3: 61.2% versus 40.3%, *p*<0.001, 95% CI [15.48–26.43]) and remained significantly higher for the entire treatment period. In molidustat‐treated groups HCT exceeded 60% on Day 21 (Group 2) and Day 14 (Group 3), respectively. Mean HCT in molidustat‐treated cats returned to levels within reference range (29–45%) on Day 56 and were numerically comparable to placebo group levels from Day 70 onwards. Red blood cell count and hemoglobin concentrations followed a similar pattern as HCT. Mean EPO concentrations were significantly higher following molidustat administrations in treated groups on all assessment days. Molidustat treatments were well tolerated.

**conclusions and clinical importance:** Remarkable erythropoietic effects were demonstrated after daily administrations of molidustat to healthy cats potentially offering a treatment option for cats with anemia.

## 16 ESOMEPRAZOLE ALTERS NEOPLASTIC CANINE MAST CELL STRUCTURE, VIABILITY, AND FUNCTION

### 16.1 **Emily N. Gould** ^1^; Elizabeth Lennon^2^, DVM, PhD, DACVIM (SAIM); Joseph Szule^3^, PhD; Heather Wilson‐Robles^4^ , DVM, DACVIM (Oncology); Joerg Steiner^1^; M. Katherine Tolbert^1,4^, DVM, PhD, DACVIM (SAIM)

#### 16.1.1 ^1^Gastrointestinal Laboratory, College of Veterinary Medicine, Texas A&M University; ^2^School of Veterinary Medicine, University of Pennsylvania; ^3^Assistant Professor, Department of Veterinary Pathobiology, College of Veterinary Medicine, Texas A&M University; ^4^Associate Professor, Veterinary Small Animal Clinical Sciences, College of Veterinary Medicine, Texas A&M University

**background:** Acid suppressant drugs are commonly used in dogs with either acid‐related gastrointestinal disorders and/or mast cell tumors. Studies in humans suggest that acid suppressants possess potentially beneficial immunomodulatory and cytotoxic properties that have yet to be investigated in companion animals.

**objectives:** To evaluate and compare the effects of esomeprazole, famotidine, and vehicle‐treatment on mast cell (MC) ultrastructure, viability, and function in vitro, using both healthy and neoplastic MCs.

**methods:** Both healthy (i.e., murine bone marrow derived [BMMC]) and neoplastic (i.e., human LAD2, canine C2 and BR) MC models were represented. Rat basophilic leukemic and canine B cell lymphoma cells served as granulocytic and agranulocytic controls, respectively. Differences in MC ultrastructure were assessed via light and transmission electron microscopy and MC viability was assessed using colorimetric assays (i.e., MTS) and flow cytometry. Changes in MC function were assessed via quantification of β‐hexosaminidase release, indicative of degranulation. All experiments were performed in triplicate fashion, with Shapiro‐Wilk, One‐Way ANOVA and Dunnett's post hoc tests used for analysis.

**results:** Only esomeprazole‐treated MCs of all lines exhibited qualitative and quantitative alterations in ultrastructure (i.e., increased cytoplasmic vacuolization, compromise of cell membrane), increased apoptosis, and altered degranulation responses. Esomeprazole was cytotoxic to all canine MCs (*P*<0.001), but not to canine B cell lymphoma cells (*P*>0.900).

**conclusions and clinical importance:** Esomeprazole, but not famotidine, is cytotoxic to MCs, causing changes in structure and function. Treatment effects on granulocytes were pronounced, but those on canine lymphoma cells were not.

‐

## 17 DECREASED DUODENAL ENTEROCYTE HEIGHT AND WIDTH IS A HALLMARK OF CHRONIC ENTEROPATHY IN DOGS

### 17.1 **Karin Allenspach** ^1^; David Díaz‐Regañón^2^ , DVM; Vojtech Gabriel^3^, DVM; Dongjie Liu^4^, BS; Vanessa Livania^5^, BS; David Meyerholz^6^, DVM, PhD, DACVP; Jonathan Mochel^7^, DVM, MS, PhD, DECVPT

#### 17.1.1 ^1^Iowa State University; ^2^Visiting PhD student, Veterinary Clinical Sciences, Iowa State University; ^3^PhD student, Veterinary Biomedical Sciences, Iowa State University; ^4^Rotating PhD student, Veterinary Biomedical Sciences, Iowa State University; ^5^MS student, Veterinary Biomedical Sciences, Iowa State University; ^6^Professor of Pathology, Division of Comparative Pathology, University of Iowa; ^7^Associate Professor of Pharmacology, Biomedical Sciences, Iowa State University

**abstract:** Celiac Disease (CD) shares many similarities with Chronic Enteropathies (CE) in dogs. Histology is commonly used to confirm diagnosis of CD with typical changes including decreased villous height‐crypt depth ratio alongside reduced enterocyte height and width. As recent WSAVA histology indices emphasized the importance of mucosal architectural changes over inflammatory infiltrates, we aimed to evaluate whether enterocyte height and width were altered in dogs with CE.

Sixty‐six dogs were included in the study (18 healthy adults, 19 healthy puppies, 11 dogs with CE and protein losing enteropathy (CE +PLE), and 18 dogs with CE without PLE (CE‐PLE)). Paraffin‐embedded duodenal biopsies (12 biopsies/dog on average) were digitalized using an Aperio scanner and analyzed for measurement of enterocytes with ImageJ software. Statistical analysis was performed by ANOVA and statistical significance set at *α*: 0.05.

Enterocyte height was significantly reduced in dogs with CE+PLE (21.34±7.01 μm) as compared to dogs with CE‐PLE (25.08±7.92 μm) and healthy adult dogs (25.02±8.84 μm). The smallest height values were reported in healthy puppies (17.04±4.79 μm). In addition, both, dogs with CE+PLE (4.94±1.20 μm) and CE‐PLE (4.61±1.43 μm) had reduced enterocyte width when compared with healthy adult dogs (5.54±1.53 μm) and healthy puppies (5.39±1.46 μm).

In conclusion, these data suggest that enterocyte height/width are significantly decreased in the duodenum of dogs with CE, consistent with previous observations in patients with CD. These findings further confirm the presence of significant architectural changes in the intestinal mucosa of dogs with CE.

## 18 ONCE‐DAILY INSULIN GLARGINE 300 U/ML FOR THE TREATMENT OF CANINE DIABETES MELLITUS

### 18.1 **Chen Gilor** ^1^; Federico Fracassi^2^, DVM, PhD, DECVIM (SAIM); Alisa Berg^1^, DVM; Aria Guarino^3^, DVM, DACVIM; Antonio Tardo^2^, DVM, DECVIM (SAIM); Linda Fleeman^4^, BVSc, PhD, MANZCVS

#### 18.1.1 ^1^University of Florida; ^2^University of Bologna; ^3^BluePearl Pet Hospital; ^4^Animal Diabetes Australia

**background:** In purpose‐bred dogs, insulin glargine 300 U/ml (Toujeo®) has a long and peakless action suitable for use as a basal insulin. There are currently no studies on its use in client‐owned diabetic dogs.

**objectives:** To describe clinical outcomes in a cohort of client‐owned dogs treated with Toujeo®.

**animals:** Fifty‐eight client‐owned diabetic dogs.

**methods:** Multi‐institutional study, including newly diagnosed dogs and dogs previously treated with other insulin formulations. Initial Toujeo® dose was 0.5 U/kg q 24 h for newly diagnosed dogs and (median [range]) 0.8 U/kg (0.2–2.5) q 24 h for the remainder. Clinical signs and standardized assessment of continuous glucose monitoring data guided dose adjustments and final categorization into level of glycemic control. Data are presented as median (range).

**results:** Thirty‐one dogs had good‐excellent glycemic control on q 24 h (1.9 U/kg [0.2–5.2]), of which 2 required additional meal‐time bolus insulin. Twenty‐seven dogs were switched to q 12 h Toujeo® (total dose=1.9 U/kg/d [0.6–5.0]). Of these, 23 had good‐excellent control (2 with the addition of meal‐time bolus insulin) and 4 had poor‐moderate control. Only one dog (treated q 12 h) experienced clinical hypoglycemia. Only 1 dog required <0.5 U/kg/daily.

**conclusions:** Overall, dogs were well controlled on Toujeo®, with half of them (29/58) maintained on once‐daily injections with no need for a meal‐time bolus insulin. Clinicians should be aware of the large dose range required to achieve good control and that overall, a higher dose of Toujeo® is required compared to previously described doses for other insulin formulations, suggesting lower potency of this insulin in dogs.

## 19 EQUINE

## 20 RISK FACTORS ASSOCIATED WITH AN EQUINE CORONAVIRUS OUTBREAK AT A SADDLEBRED FARM IN NORTH CAROLINA

### 20.1 **Kate L.** **Hepworth‐Warren** ^1^ ; Sara Erwin^1^; James Talbot^2^, DVM; Caroline Moore^3^; Spencer Williams^4^; Miriam Johnson^4^; Kimberly Young^1^, DVM; Carol Woodlief^4^, DVM; Jennifer Haugland^5^; Michael Neault^6^, DVM; Jim Trybus^7^, DVM, DAVP; Anthony Blikslager^8^, DVM, PhD, DACVS

#### 20.1.1 ^1^College of Veterinary Medicine, North Carolina State University; ^2^Carolina Equine Hospital; ^3^Jacksonville Equine Associates; ^4^North Carolina Department of Agriculture & Consumer Services; ^5^Rollins Animal Disease Diagnostic Laboratory; ^6^Clemson University; ^7^North Carolina Veterinary Diagnostic Laboratory System; ^8^Department Head, Department of Clinical Sciences, College of Veterinary Medicine, North Carolina State University

**background:** Equine coronavirus (ECoV) leads to outbreaks with variable morbidity and mortality. Few previous reports of risk factors for infection are available.

**objectives:** To describe unique clinical findings and risk factors for infection and development of clinical disease with ECoV.

**animals:** 135 horses from farm affected by ECoV outbreak.

**methods:** Data obtained for each horse included age, breed, gender, activity level, housing, and feed at the onset of the outbreak. Factors were evaluated for assessment of risk of infection using simple logistic regression or Fisher's exact test. Significance was set at *p*≤0.05.

**results/findings:** Forty‐three of 135 horses (31.8%) were positive on fecal PCR for ECoV, and 17 horses (12.6%) developed clinical signs. Colic occurred in 46.2% of affected animals. Three horses had small colon impactions, 2 of which required surgical intervention. Significant risk factors for having positive PCR results included presence of clinical signs (OR 56, 95% CI 8.298–594.6), being primarily stalled (OR 167.1, 95% CI 26.41–1719), housing next to a positive horse (OR 7.5, 95% CI 3.100–18.97), being in work (OR 26.9, 95% CI 4.573–281.9), being fed a ration of hay vs. ad libitum (OR 418, 95% CI 36.83–4438), being fed alfalfa hay (OR 418, 95% CI 36.83–4438) and levothyroxine supplementation (OR 7, 95% CI 2.723–18.68).

**conclusions and clinical importance:** This report describes risk factors for ECoV infection, including a series of factors associated with intensive management of show horses. Clinicians should be aware that clinical signs of ECoV vary and can include severe colic.

## 21 VOLUNTARY SURVEILLANCE PROGRAM FOR EQUINE INFLUENZA VIRUS IN THE UNITED STATES FROM 2008–2021

### 21.1 **Fairfield T. Bain** ^1^; Duane Chappell^2^, DVM; Bryant Craig^1^, DVM; Kaitlyn James^3^, PhD; Chrissie Schneider^1^, DVM, DABVP (Equine); Wendy Vaala^4^, VMD, DACVIM (LAIM)

#### 21.1.1 ^1^Merck Animal Health; ^2^Associate Director, Equine Professional Services, Merck Animal Health; ^3^Independent contractor, Merck Animal Health; ^4^Director, Strategic Development and Innovation, Merck Animal Health

**background:** Equine influenza virus (EIV) is an enzootic cause of respiratory disease in equids as documented in studies from most of the world. Horses with EIV infection have consistent clinical signs of fever, nasal discharge, and cough. Current knowledge of patterns of EIV is relevant to the Internist.

**objectives:** This study evaluated epidemiologic and clinical features of horses with signs of infectious respiratory disease sampled through a biosurveillance program.

**animals:** Veterinarians in practice submitted samples from sentinel horses showing signs compatible with infectious respiratory disease. A total of 9,740 horses, mules, and donkeys were included.

**methods:** Samples obtained from December 2008–June 2021 were analyzed. A questionnaire was used to collect clinical history and case information. Whole blood and nasal secretions were sampled from horses with fever (>101.5°F) and signs of acute upper respiratory disease. Samples were evaluated with real‐time PCR for equine herpesvirus type 1 and 4, equine influenza virus, equine rhinitis virus A & B and *Streptococcus equi* subsp. *equi*.

**results:** 966 equids tested positive for EIV yielding a 9.9% positivity rate. The study documented comparable incidence to equine herpesvirus type 4, age of incidence mostly commonly in 1‐ to 9‐year‐old equids, primary seasonality in winter to early spring (though identified throughout the year) and occurrence of prior travel in positive individuals.

**conclusions:** This analysis supports the importance of EIV as a frequent pathogen associated with equine upper respiratory disease in the United States and provides contemporary data from EIV detection in the United States.

## 22 ANTIBIOGRAM USE TO INFORM ANTIMICROBIAL SELECTION IN EQUINE PRACTICE

### 22.1 **Sharanne L. Raidal** ^1^; Surita du Preez^2^, BVSc, MANZCVS, DECEIM, DVStud; Darren Trott^3^, BSc (Hons), BVMS (Hons), PhD

#### 22.1.1 ^1^School of Agricultural, Environmental and Veterinary Sciences, Charles Sturt University; ^2^Senior Lecturer in Equine Medicine, Equine Health and Performance Centre, The University of Adelaide; ^3^Professor of Veterinary Microbiology, Australian Centre for Antimicrobial Resistance Ecology, The University of Adelaide

**background:** The identification of bacterial species and their susceptibilities permits targeted antimicrobial treatment, has been shown to improve the outcome of clinical cases, and may reduce the emergence of resistance to available antimicrobials. Recommendations for collation of cumulative antimicrobial susceptibility tables, known as antibiograms, have been developed and utilised in human medicine, but have to date had limited use in equine practice.

**hypothesis/objectives:** Development of a stratified antibiogram will expediate review of sensitivity profiles, facilitate drug selection, and is likely to improve antimicrobial stewardship in equine clinical settings.

**methods:** Recommendations for antibiogram development based on bacterial culture and sensitivity results were reviewed, refined, and implemented for clinical submissions to veterinary diagnostic laboratories at Charles Sturt University and the University of Adelaide between 2010 and 2020.

**results:** Data from 1161 isolates demonstrated differences in bacterial species identified and antimicrobial sensitivity of isolates attributable to sample type, location, and between hospital based and ambulatory practices. Increased resistance was observed in *Staphylococcus* and *Pseudomonas* spp. isolated from samples collected in the later part of the study (2016–2020), compared to isolates from early samples (2010–2015). Enterobacterales isolates, *Enterococcus* and *Pseudomonas* spp. were more likely to demonstrate multiple (65.7%, 77.8% and 92.1%, respectively) and extensive (27.4%, 47.5% and 61.8%, respectively) drug resistance profiles.

**conclusions and clinical importance:** The antibiogram proved useful for rapidly filtering available results to facilitate antimicrobial selection, and for evaluation of data subsets. Future studies should evaluate the impact of such measures on clinical outcomes.

‐

## 23 PHARMACOKINETICS AND EX VIVO PHARMACODYNAMICS OF THE NOVEL ANTI‐INFLAMMATORY DRUG, DORAMAPIMOD, IN HORSES

### 23.1 **Jenni R. Bauquier** ^1^; Russell Pickford^2^; Simon Bailey^1^

#### 23.1.1 ^1^University of Melbourne; ^2^University of New South Wales

**background:** Novel anti‐inflammatory drugs are needed to better manage systemic inflammation in horses. Doramapimod has potent anti‐inflammatory effects in horses, but pharmacological studies in horses are lacking.

**hypothesis/objectives:** To determine the pharmacokinetics and ex vivo pharmacodynamics of doramapimod in horses.

**animals:** Five healthy Standardbred horses.

**methods:** A 0.5 mg/kg bolus dose of doramapimod was administered intravenously to all horses. Blood samples were obtained at baseline and serially over 72 hours. Also, at 6 timepoints peripheral blood leukocytes were isolated, washed, resuspended in 1 ml saline; then frozen. Plasma and leukocyte doramapimod concentrations were assayed by liquid chromatography‐mass spectrometry. Further, aliquots of whole blood from each time point were stimulated with lipopolysaccharide (0‐1 μg/ml) and supernatant concentrations of TNF‐α and IL‐1β were measured by multiplex ELISA.

**results:** Plasma elimination half‐life of doramapimod was 3.88±1.05 hours, with concentrations close to the limit of detection by 24 hours. However, doramapimod was still detectable in leukocytes up to 48 hours after administration (0.21±0.04 and 0.05±0.02 ng/10^7^ cells at 24 and 48 hours, respectively). Inhibition of non‐lipopolysaccharide‐induced pro‐inflammatory cytokine response in whole blood was also evident at 48 hours (61.6±23.1% and 47.5±29.4% inhibition of TNF‐α and IL‐1β production respectively).

**conclusions and clinical importance:** Doramapimod appears to bind and accumulate within leukocytes, producing measurable effects well after minimal plasma concentrations are reached. This ‘effect site pharmacokinetics’ suggests that, for the treatment of acute inflammatory conditions where leukocyte activation is targeted (e.g., sepsis), a dosing interval of 24 hours may be suitable despite the short plasma half‐life.

## 24 FEASIBILITY OF A POINT‐OF‐CARE ULTRASOUND PROTOCOL FOR CARDIORESPIRATORY EVALUATION IN HORSES

### 24.1 **Kari Bevevino** ^1^; Noah Cohen^2^; Sonya Gordon^2^; Cris Navas^3^

#### 24.1.1 ^1^Roaring Fork Equine Medical Center; ^2^Texas A&M; ^3^University of Pennsylvania

**background:** A point‐of‐care ultrasound (POCUS) protocol for sonographic evaluation of the cardiorespiratory system in horses does not exist.

**hypothesis/objectives:** 1) To describe the windows of CRASH, a POCUS protocol for cardiorespiratory assessment of the horse; 2) To determine the proportion of acoustic windows that can be acquired by a sonographer in training; 3) To determine the time required to complete the protocol for specific groups of horses; 4) To describe the sonographic abnormalities detected in horses presented with cardiovascular, respiratory, or systemic disease.

**animals:** Twenty‐seven healthy horses, 14 horses competing in athletic events and 121 horses with clinical disease.

**methods:** A pocket‐sized ultrasound device was used to acquire 7 sonographic windows to assess the cardiorespiratory system. The duration of the examination was timed, and all images were evaluated for diagnostic quality. Horses with clinical disease had images evaluated for the presence of abnormalities.

**results:** The CRASH protocol could be performed in healthy and diseased horses in a hospital, barn, and competition settings within 5.5 to 6.9 minutes on average. Thoracic windows were obtained most consistently, followed by right parasternal long axis echocardiographic windows. Abnormalities detected most frequently with the CRASH protocol were pleural fluid, lung consolidation, B lines, and moderate‐to‐severe left‐sided heart disease.

**conclusions:** The designed CRASH protocol is feasible for use in various groups of horses and can be completed in a timely manner and frequently identifies sonographic abnormalities. The diagnostic accuracy and potential of the CRASH protocol to improve veterinary care of horses merits further systematic evaluation.

## 25 EFFECTS OF NON‐INVASIVE VENTILATION ON RESPIRATORY FUNCTION AND LUNG VOLUME IN FOALS

### 25.1 **Sharanne L. Raidal** ^1^; Melanie Catanchin^2^, BVSc (Hons); Muriel Sacks^3^; Ann Carstens^4^; Chris Quinn^4^; Martina Mosing^3^

#### 25.1.1 ^1^School of Agricultural, Environmental and Veterinary Sciences, Charles Sturt University; ^2^Lecturer in Veterinary Anaesthesiology, Veterinary Clinical Centre, Charles Sturt University; ^3^Murdoch University; ^4^Charles Sturt University

**background:** Respiratory disease and prolonged recumbency impair ventilation in foals due to lung collapse. Non‐invasive ventilation (NIV) is used in many intensive care settings, and may offer improved respiratory support to foals.

**hypothesis/objectives:** NIV using pressure support mode with increasing positive end‐expiratory pressure (PEEP) would be associated with improved respiratory function in sedated recumbent foals.

**animals:** Six healthy foals from a university teaching herd.

**methods:** A prospective, randomised cross‐over study evaluated NIV in sedated foals with low (2 and 4 cm H_2_O) and high PEEP (4, 7 and 10 cm H_2_O) protocols. Spirometry and arterial blood gas analysis were used to assess respiratory function and gas exchange, respectively. Physiological dead space (VD_phys_) and volume of CO_2_ exhaled per breath (VCO_2_/br) were determined using volumetric capnography. Pulmonary aeration and lung volume were determined by computer tomography.

**results:** NIV was associated with decreased respiratory rate and increased tidal volume (both *P*<0.001); no change was observed in VD_phys_. Arterial oxygenation increased (*P*=0.011) with higher PEEP values, and no changes in carbon dioxide partial pressures were observed. VCO_2_/br, pulmonary aeration and lung volume increased in a dose‐dependent manner with higher PEEP settings.

**conclusions and clinical importance:** NIV at high PEEP levels improved respiratory function in sedated foals, increased ventilated lung area and enhanced CO_2_ elimination per breath without alveolar overdistension. NIV should be considered for respiratory support of neonates with impaired respiratory function.

## 26 FOOD ANIMAL INTERNAL MEDICINE

## 27 PROGNOSTIC INDICATORS FOR SURVIVAL OF DOWNER COWS TREATED WITH USE OF A FLOTATION TANK

### 27.1 **Chelsea Holschbach** ^1^; Simon Peek^2^, BVSc, PhD, DACVIM (LAIM); Sarah Raabis^3^, DVM, PhD, DACVIM (LAIM)

#### 27.1.1 ^1^UW School of Veterinary Medicine; ^2^Professor, UW School of Veterinary Medicine; ^3^Postdoctoral fellow, Pathobiological sciences, UW School of Veterinary Medicine

**background:** Non‐ambulatory cattle present a diagnostic and therapeutic challenge and raise animal welfare concerns. For valuable individual animals, treatment by flotation therapy is an option, but concerns of cost versus return exist. Prognostic indicators for survival are needed to help guide treatment decisions.

**hypothesis/objectives:** To evaluate historical and clinical variables assessed during hospitalization as prognostic indicators for survival in recumbent cattle that underwent flotation treatment in a referral hospital.

**animals:** 190 non‐ambulatory dairy cattle referred for flotation treatment.

**methods:** Retrospective case‐series; medical records were analyzed from dairy cattle undergoing flotation between 2000–2020. Univariable and multivariable logistic regression analyses were performed to assess the association of clinical variables with survival to discharge.

**results:** Eighty‐nine of 190 (47%) recumbent cattle survived. Cattle that were unable to walk out of the tank after their first float session were 0.10 (95% CI, 0.03, 0.25) times less likely to survive compared to cattle that could. Inappetent cattle were 0.21 (95% CI, 0.07, 0.58) times less likely to survive compared to cattle with normal appetites. Cattle that were recumbent due to calving paralysis or from metabolic derangements were 10.53 (95% CI, 1.22, 110.75) and 22.02 (95% CI, 4.87, 122.22) times more likely to survive, respectively, compared to those recumbent due to coxofemoral luxation.

**conclusions and clinical importance:** Diagnosis, behavioral factors during first float session, and appetite are associated with outcome in non‐ambulatory dairy cattle treated by flotation. These findings can be used to determine likely case outcome and assist with decisions concerning treatment, referral, or euthanasia.

## 28 EFFECTS OF BLOOD CONTAMINATION ON TOTAL NUCLEATED CELL COUNTS AND PROTEIN CONCENTRATIONS IN BOVINE CSF

### 28.1 **Maria** **Puerto‐Parada** ^1^ ; Gilles Fecteau^2^, DVM, DACVIM‐LA; Juan Carlos Arango‐Sabogal^3^ , MV, PhD; Christian Bédard^3^, DMV, MSc, DACVP

#### 28.1.1 ^1^Université de Montréal; ^2^Professor, Clinical Sciences, Université de Montréal; ^3^Professor, Pathology and Microbiology, Université de Montréal

Analysis of cerebrospinal fluid (CSF) is a useful procedure for determining a diagnosis and prognosis in neurologic patients. Blood contamination during CSF sampling may confound interpretation of results since total nucleated cells count (TNCC) and total protein concentration (TPC) could be falsely elevated. Accurately predicting TNCC and TPC attributable to blood contamination is difficult.

The objective was to determine the effects of blood contamination on bovine CSF TNCC and TPC using samples spiked with known dilutions of whole blood.

Cerebrospinal fluid was collected from the lumbosacral subarachnoid space in 9 clinically normal cows. For each cow, 1 μL (5.76–6.92x10^6^ RBC) of her own blood was added to 999 μL of her CSF (sample A). Next, sample A was diluted 1:2, 1:5, 1:10, 1:100 and 1:1000 (sample B to F, respectively) using CSF from the same animal (0–1050 RBC/μL). TNCC and TPC were measured for all samples (prior and after adding blood). Repeated measures ANOVA were used to assess whether there was a difference in TNCC and PC at each dilution.

Overall, there was a significant association between the amount of blood added and the TNCC and TPC obtained values (*P*<0.01). A significant difference on TNCC and TPC was observed (*P*<0.01) between CSF before addition of blood and the samples A and B. On samples C to F, TNCC and TPC were not significantly different from original sample values.

While blood contamination appears to impact the TNCC and TPC, up to 3350 RBC/μL are necessary to measure a significant difference according to this experimental design.

